# Risk factors for recurrent *Clostridium difficile* infection (CDI) hospitalization among hospitalized patients with an initial CDI episode: a retrospective cohort study

**DOI:** 10.1186/1471-2334-14-306

**Published:** 2014-06-04

**Authors:** Marya D Zilberberg, Kimberly Reske, Margaret Olsen, Yan Yan, Erik R Dubberke

**Affiliations:** 1 University of Massachusetts, Amherst, MA, USA; 2 EviMed Research Group, LLC, PO Box 303, Goshen, MA, USA; 3 Washington University School of Medicine, 660 S. Euclid Ave, St. Louis, MO 63110, USA

**Keywords:** *C. difficile*, Risk factors, Recurrence

## Abstract

**Background:**

Recurrent *Clostridium difficile* infection (rCDI) is observed in up to 25% of patients with an initial CDI episode (iCDI). We assessed risk factors for rCDI among patients hospitalized with iCDI.

**Methods:**

We performed a retrospective cohort study at Barnes-Jewish Hospital from 1/1/03 to 12/31/09. iCDI was defined as a positive toxin assay for *C. difficile* with no CDI in previous 60 days, and rCDI as a repeat positive toxin ≤42 days of stopping iCDI treatment. Three demographic, 13 chronic and 12 acute disease characteristics, and 21 processes of care were assessed for association with rCDI. Cox modeling identified independent risk factors for rCDI.

**Results:**

425 (10.1%) of 4,200 patients enrolled developed rCDI. Of the eight risk factors for rCDI on multivariate analyses, the strongest three were 1) high-risk antimicrobials following completion of iCDI treatment (HR 2.95, 95% CI 2.25-3.86), 2) community-onset healthcare-associated iCDI (HR 1.80, 95% CI 1.41-2.29) and 3) fluoroquinolones after completion of iCDI treatment (HR 1.56, 95% CI 1.63-2.08). Other risk factors included gastric acid suppression, ≥2 hospitalizations within prior 60 days, age, and IV vancomycin after iCDI treatment ended.

**Conclusions:**

The rCDI rate was 10.1%. Recognizing such modifiable risk factors as certain antimicrobial treatments and gastric acid suppression may help optimize prevention efforts.

## Background

Over the past decade *Clostridium difficile* infection (CDI) has increased in both frequency and severity in the US and abroad. A study from Quebec identified a 5-fold rise in the incidence of hospitalizations with CDI over 13 years, accompanied by a doubling in the risk of complicated disease [1]. Similarly, multiple US-based studies have reported a more-than-doubling of hospitalizations with a CDI diagnosis between 2000 and 2005 [2,3]. These numbers have continued to rise through 2009, albeit less rapidly [4]. Much of this growth is thought to be due to the recent emergence of the hypervirulent epidemic strain of *C. difficile*, BI/NAP1/027. A fluoroquinolone-resistant toxin overproducer, this strain has now been detected in most of the states in the US, in North America, Europe and beyond [5].

One of the most challenging aspects of CDI is its propensity to recur. Both metronidazole and vancomycin, first-line therapies recommended in the joint evidence-based practice guideline from the Society of Healthcare Epidemiology of America (SHEA) and Infectious Diseases Society of America (IDSA), have exhibited unacceptably high rates of recurrence [6]. Indeed, a recent meta-analysis has found that CDI recurs in 13% – 50% of all patients after an initial episode, and in the setting of a randomized controlled trial, the recurrence rate was 25% [7-9].

Recurrent CDI (rCDI) is a cause of much morbidity, and its economic impact is likely substantial. Several studies have identified important risk factors for rCDI, including advanced age, chronic renal insufficiency, elevated white blood cell count, low serum albumin, use of proton pump inhibitors (PPI), and continued use of systemic antimicrobials during the initial CDI episode (iCDI) [7,10-13]. However, a meta-analysis identified major gaps in our understanding of the risk factors for CDI recurrence [7]. Although the authors found concomitant antimicrobials, gastric acid suppressants and older age to be strongly predictive of rCDI, other factors, including iCDI treatment and specific non-CDI antimicrobials, could not be evaluated adequately due to the lack of robust data. Additionally, most studies have focused on the factors immediately preceding rCDI onset, ignoring the possibility that factors present at or near the onset of the iCDI episode may also impact this risk. In fact, recent data suggest that the burden of community-onset healthcare-facility associated (CO-HCFA) CDI is much higher than previously appreciated, and poses an additional risk pool for inpatient exposure [14,15]. Since CO-HCFA implies an ongoing exposure to the healthcare system, it may itself be a marker for a recurrence.

A precise understanding of who is likely to recur is an important clinical question for two reasons. First, if there are modifiable exposures that increase this risk, knowing what they are may aid clinicians in avoiding them. Second, if patient characteristics not subject to modification predispose to rCDI, recognizing them may help target preventive measures more effectively. In order to define more fully the risk factors for rCDI, we conducted a single center retrospective cohort analysis among patients hospitalized with an iCDI episode.

## Methods

This study was approved by the Washington University Institutional Review Board, and its conduct was in compliance with the Helsinki Declaration.

### Cohort definition

We conducted a retrospective cohort study of all adult (age ≥18 years) patients with an inpatient episode of iCDI at Barnes-Jewish Hospital (BJH) between January 1, 2003, and December 31, 2009. An episode of CDI was defined as a positive toxin assay (C. DIFFICILE TOX A/B II from Techlab, Blacksburg, VA, USA) for *C. difficile*. Because the hospital laboratory performs a test for *C. difficile* only if the treating physician suspects CDI and if the stool is unformed, all patients with positive toxin results were considered to be CDI case patients. The first episode of CDI during the study period in the absence of any CDI in the prior 60 days was defined as the iCDI, and patients were included only once. Patients were excluded if they died during or were discharged to hospice from the iCDI hospitalization.

All included patients were followed for 42 days from the date of the end of iCDI treatment or until rCDI onset, defined as a repeat positive toxin within this time frame. Initial CDI cases were categorized according to published surveillance definitions as community-onset healthcare facility-associated (CO-HCFA) (indeterminate CDI cases were grouped with CO-HCFA), healthcare facility-onset (HCFO), and community-associated (CA) [16].

### Data sources

Demographic and clinical data were derived from BJH Medical Informatics databases and the BJH electronic medical records. The data available from the Informatics databases included *C. difficile* toxin assay results and date of stool collection; patient demographics; dates of admission and discharge; discharge disposition; admission location; ICD-9-CM diagnosis (used to define underlying comorbidities in the year prior and during the index hospitalization) and procedure (assessed only during the index hospitalization) codes; dates of ICU stays; start and stop dates of all inpatient CDI treatments, gastric acid suppressors and antimicrobials; and white blood cell count, hemoglobin, serum creatinine, and serum albumin levels on admission and at the time of positive *C. difficile* toxin assays from the index admission and all readmissions in the 42 days after iCDI treatment end. The BJH medical records included data on antimicrobials and CDI treatments the patient received as an outpatient within the BJH system, and whether a readmission was for CDI. In addition, admission and discharge summaries were reviewed for all included hospitalizations to help determine whether the patient had a history of CDI at another healthcare facility or as an outpatient.

### Statistical analyses

The exposure interval was divided into three periods: 1) from hospital admission until diagnosis of iCDI, 2) from the time of diagnosis of iCDI until the end of its treatment, and 3) from the end of iCDI treatment until the onset of recurrence or until the end of the 42-day monitoring period for recurrence. We compared patients with rCDI to those without rCDI based on their characteristics in these time periods. Cox proportional hazards modeling was used to determine variables associated with at least one episode of rCDI on univariate analysis. Antimicrobials were categorized based on association with CDI as high-risk (cephalosporins, aminopenicillins, and clindamycin), low-risk (aminoglycosides, beta lactamase inhibitors, carbepenems, daptomycin, doxycycline, linezolid, macrolides, rifampin, rifaximin, and tigecycline), fluoroquinolones (>90% was ciprofloxacin), and intravenous vancomycin [17,18]. Gastric acid suppressors (histamine receptor 2 blockers [HR2B] and proton pump inhibitors [PPI]), choice and duration of iCDI treatment, and iCDI severity, as defined by the SHEA/IDSA Clinical Practice Guidelines for CDI, were also assessed as potential risk factors for rCDI [6].

We employed extended Cox proportional hazards modeling to determine independent risk factors for at least one episode of rCDI, with variable selection according to the methodology of Hosmer-Lemeshow [19]. Variables eligible for inclusion in the multivariable models were those associated with increased risk of rCDI from the literature or those with clinical or biologic plausibility, and those with p-values <0.20 in the univariate analyses. Antimicrobial exposures from the end of CDI treatment until rCDI or 42 days were analyzed as time-dependent variables. Backward stepwise selection was used to arrive at the best-fitting and most parsimonious model. All relevant 2-way interactions were tested after selection of the main effects, and included in the final models only if they were significant at the alpha ≤0.05. The proportional hazards assumption was verified by assessing the parallel nature of curves in log-log plots. The appropriate functional formats of continuous variables were determined by examining nonparametric regression (smoothing) plots with a restricted cubic spline function. To facilitate interpretation of results, the hazard ratios for the piecewise linear spline variable (fluoroquinolone exposure while on CDI treatment) compared the hazards of developing CDI for values between the 75th and the 25th percentiles of the variable [20]. To assess the importance of time dependency for antimicrobial exposures that occur after CDI treatment, these exposures included in the final model were also analyzed in a time-independent fashion.

All analyses were performed in SAS version 9.3 (SAS Institute, Cary, NC) and R (R Foundation, Vienna, Austria) [21]. All statistical testing was two-tailed with significance set at the alpha level ≤ 0.05.

## Results

Among the 4,200 patients enrolled with iCDI, 425 (10.1%) had at least one rCDI identified. Those with a recurrence were older (median age 64.8, range 18.3 – 98.2, vs. 61.6, range 18.0 – 102.4), and had a greater comorbidity burden, as evidenced by the higher Charlson score than those without (Table 1). Of the specific comorbidities, diabetes mellitus was more prevalent in the rCDI group than in the group without rCDI. Notably, prior exposure to the healthcare system was more likely among those with rCDI than those without. Persons with rCDI were nearly twice as likely to fit the surveillance definition for CO-HCFA CDI during their initial episode (39%) as those without rCDI (22%, p < 0.001). Consistent with this, patients with rCDI had a higher frequency of at least one inpatient admission within 60 days prior to the iCDI episode (53% vs. 39%, p < 0.001), as well as a higher risk for multiple recent hospitalizations than those without rCDI (Table 1). The iCDI admission laboratory data did not differ substantively between the groups.

**Table 1:** Patient characteristics and treatments at hospital admission involving the initial CDI episode

**Patient characteristics**	**Patients who developed rCDI**	**Patients who did not develop rCDI**	**Hazard ratio**
	**(n = 425)**	**(n = 3775)**	**95% CI**
Demographics			
Age, years (median[range])	64.8 (18.3 – 98.2)	61.6 (18.0 –102.4)	1.01 (1.01 – 1.02)
Gender: female	210 (49)	1824 (48)	1.05 (0.87 – 1.27)
Comorbidities^a^			
Myocardial infarction	40 (9)	328 (9)	1.12 (0.81 – 1.55)
Congestive heart failure	108 (25)	854 (23)	1.23 (0.99 – 1.53)
Peripheral vascular disease	34 (8)	269 (7)	1.13 (0.79 – 1.60)
Cerebrovascular disease	41 (10)	256 (7)	1.51 (1.10 – 2.09)
Chronic renal failure	21 (5)	190 (5)	0.98 (0.64 – 1.53)
Dementia	5 (1)	23 (1)	1.83 (0.76 – 4.41)
Chronic obstructive pulmonary disease	116 (27)	912 (24)	1.18 (0.95 – 1.46)
Rheumatologic disease	18 (4)	146 (4)	1.11 (0.69 – 1.78)
Peptic ulcer disease	20 (5)	154 (4)	1.18 (0.75 – 1.85)
Mild liver disease	17 (4)	201 (5)	0.81 (0.50 – 1.32)
Moderate to severe liver disease	12 (3)	134 (4)	0.86 (0.48 – 1.53)
Diabetes	135 (32)	975 (26)	1.32 (1.08 – 1.62)
Paraplegia or hemiplegia	12 (3)	78 (2)	1.40 (0.79 – 2.45)
Any malignancy (excluding leukemia/lymphoma)	83 (20)	770 (20)	0.99 (0.78 – 1.25)
Leukemia or lymphoma	78 (18)	660 (18)	1.05 (0.82 – 1.34)
Metastatic solid tumor	56 (13)	449 (12)	1.19 (0.90 – 1.58)
HIV/AIDS	10 (2)	66 (2)	1.30 (0.70 – 2.44)
Charlson composite score			
0-2	223 (53)	2182 (58)	Ref
3-5	117 (28)	922 (24)	1.27 (1.01 – 1.59)
> = 6	85(20)	671 (18)	1.32 (1.03 – 1.69)
Case status^b^			
HCFO/HCFA	203 (48)	2332 (62)	Ref
CA or unknown	57 (13)	597 (16)	1.07 (0.79 – 1.43)
CO/HCFA, indeterminate, or non- BJH HCFA	165 (39)	846 (22)	2.17 (1.76 – 2.66)
Admitted from another healthcare facility	109 (26)	1019 (27)	0.97 (0.78 – 1.21)
Number of inpatient admissions in previous 60 days			
0	200 (47)	2313 (61)	Ref
1	150 (35)	1021 (27)	1.70 (1.37 – 2.10)
2+	75 (18)	441 (12)	1.96 (1.50 – 2.55)
Baseline laboratory data^c^			
Low albumin at admission	52 (12)	522 (14)	0.94 (0.70 – 1.25)
Low WBC at admission	44 (10)	420 (11)	0.92 (0.68 – 1.26)
High WBC at admission	236 (56)	2122 (56)	0.86 (0.66 – 1.12)
Low hemoglobin at admission	182 (43)	1572 (42)	1.09 (0.90 – 1.32)
High creatinine at admission	108 (25)	947 (25)	1.04 (0.84 – 1.30)
Low creatinine clearance	218 (51)	1636 (43)	1.43 (1.18 – 1.73)

rCDI = recurrent *C. difficile* infection.

^a^Comorbidities diagnosed within previous 1 year (identified by ICD-9-CM diagnosis codes.

^b^HCFO = health care facility onset; HCFA = healthcare facility-associated; CA = community acquired; CO = community onset. Case she status for 6 patients’ was unknown: 1 among those who developed rCDI and 5 among those who did not.

^c^The following threshold values defined “high” and “low” levels: albumin <2.5 g/dL; WBC low <3.8*10^3^/mm^3^; WBC high >9.8*10^3^/mm^3^; hemoglobin <10.0 g/dL; creatinine >1.5 ug/dL; creatinine clearance <70 mL/min. WBC = white blood cells.

There were several differences between patients with and those without rCDI with respect to exposure to medications known to raise the risk of CDI (Table 2). Nearly ¾ of all patients in each group were on at least one antimicrobial at the onset of their iCDI. There were no differences in antibiotics considered to be “high-risk” for causing CDI between the rCDI and non-rCDI groups. Conversely, antimicrobials designated as “low-risk” for CDI were used with lower frequency in patients with (22%) compared to those without rCDI (28%, p = 0.02). Fluoroquinolone treatment was more prevalent during the iCDI onset among those with rCDI (28%) than those without (23%, p = 0.02). Furthermore, patients with rCDI who used gastric acid suppressors were nearly twice as likely as those without to be started on one within 24 hours of the iCDI diagnosis (Table 2).

**Table 2:** Processes of care at the onset of and treatment for the initial CDI hospitalization

**Patient characteristics**	**Patients who developed rCDI**	**Patients who did not develop rCDI**	**Hazard ratio**
	**(n = 425)**	**(n = 3775)**	**95% CI**
Laboratory results iCDI onset			
Low albumin	50 (12)	548 (15)	0.84 (0.63 – 1.13)
Low WBC	64 (15)	635 (17)	0.99 (0.82 – 1.20)
High WBC	247 (58)	2027 (54)	1.23 (1.01 – 1.49)
Low hemoglobin	218 (51)	1985 (53)	0.96 (0.79 – 1.16)
High creatinine	99 (23)	862 (23)	1.08 (0.86 – 1.35)
Relevant medications present at iCDI onset			
Any antimicrobial	314 (74)	2729 (72)	1.10 (0.88 – 1.36)
Low risk antimicrobial(s)^a^	95 (22)	1058 (28)	0.76 (0.60 – 0.95)
High risk antimicrobial(s)^b^	174 (41)	1490 (40)	1.07 (0.88 – 1.29)
Fluoroquinolone	120 (28)	861 (23)	1.29 (1.05 – 1.60)
IV vancomycin	130 (31)	1321 (35)	0.86 (0.70 – 1.05)
Gastric acid suppressor, any	310 (73)	2850 (76)	0.91 (0.73 – 1.12)
New gastric acid suppressor	54 (13)	255 (7)	1.87 (1.41 – 2.49)
Relevant medications received following iCDI onset			
Any antibiotic first dose after CDI	278 (65)	1622 (43)	2.47 (2.02 – 3.02)
Low risk antimicrobial(s) first dose after CDI^a^	141 (33)	710 (19)	2.09 (1.71 – 2.56)
High risk antimicrobial(s) first dose after CDI^b^	150 (35)	714 (19)	2.30 (1.89 – 2.81)
Fluoroquinolone first dose after CDI	124 (29)	703 (19)	1.69 (1.37 – 2.09)
IV vancomycin first dose after CDI	115 (27)	337 (12)	2.61 (2.11 – 3.23)
Initial CDI treatment			
Metronidazole alone	323 (76)	2841 (75)	Reference
Oral vancomycin alone	16 (4)	104 (3)	1.32(0.80 – 2.18)
Metronidazole and oral vancomycin	86 (20)	829 (22)	0.95 (0.75 – 1.20)

iCDI = initial episode of *C. difficile* infection, rCDI = recurrent *C. difficile* infection.

^a^Low risk antimicrobials included aminoglycosides, betalactamase inhibitors, carbepenems, daptomycin, doxycycline, linezolid, macrolides, penicillinase inhibitors, rifampin, rifaximin, and tigecycline.

^b^High risk antimicrobials included all cephalosporins, clindamycin, and penicillins.

Although there were no differences in the treatment regimen aimed at the iCDI episode, patients who developed rCDI were more frequently started on such high-risk medications as gastric suppressors and non-CDI antimicrobials, regardless of their designation as high- or low-risk, after the onset of iCDI (Table 2). Those with subsequent rCDI were more likely to be discharged to a healthcare facility following the iCDI hospitalization than those without rCDI, though this difference did not reach statistical significance. Following the hospitalization with iCDI, patients with a subsequent rCDI were also more likely to be readmitted to the hospital both before and/or after the end of the iCDI treatment (Table 3).

**Table 3:** Outcomes following initial CDI hospitalization

**Patient characteristics**	**Patients who developed rCDI**	**Patients who did not develop rCDI**	**Hazard ratio**	**p-value**
	**(n = 425)**	**(n = 3775)**	**95% CI**	
Discharged to a healthcare facility	130 (31)	1029 (27)	1.18 (0.96 – 1.45)	0.11
Inpatient readmission(s) before end of iCDI treatment	48 (11)	241 (6)	1.76 (1.30 – 2.37)	<.001
Inpatient readmission(s) after end of iCDI treatment	126 (30)	857 (23)	1.31 (1.07 – 1.62)	0.01

iCDI = initial episode of *C. difficile* infection, rCDI = recurrent *C. difficile* infection.

In a multivariate Cox proportional hazards model, where we examined 49 potential covariates for their impact on the risk of rCDI, eight factors emerged as predictive of a future episode of rCDI (Table 4). In addition to age, case status of iCDI designation of CO-HCFA was strongly associated with rCDI, increasing its risk by 80% (HR 1.80, 95% CI 1.41 to 2.29). In a similar vein, having had two or more inpatient hospitalization within 60 days prior to the onset of CDI was associated with an increased risk of rCDI (HR 1.40, 95% CI 1.04-1.89). A number of modifiable risk factors also predicted the risk of rCDI. They included initiation of gastric acid suppressors at the time of iCDI diagnosis (HR 1.36, 95% CI 1.004 to 1.85), and cumulative exposure to fluoroquinolones while on iCDI therapy (HR 1.45, 95% CI 1.09 to 1.41). Exposure to fluoroquinolones (HR 1.56, 95% CI 1.16 to 2.08), IV vancomycin (HR 1.45, 95% CI 1.09 to 1.92), and high-risk antimicrobials (HR 2.95, 95% CI 2.25 to 3.86) at any time *t* after the end of iCDI therapy ended also increased the risk of rCDI. Demonstrating the importance of time dependency of concomitant antimicrobials started after CDI treatment ended, when post-CDI treatment antimicrobials were modeled in a time independent fashion, their association with rCDI decreased significantly (Table 4).

**Table 4:** Cox proportional hazards multivariable model examining risk factors for recurrent CDI

**Patient characteristics**	**Antimicrobials after iCDI treatment modeled as time dependent variables**		**Antimicrobials after iCDI treatment modeled as time independent variables**	
**Risk factor**	**Hazard ratio**	**95% CI**	**Hazard ratio**	**95% CI**
*At admission to the hospital*				
CDI case status				
HO CDI	Ref		Ref	
COHCFA CDI	1.80	1.41-2.29	1.78	1.39-2.27
CA CDI	1.30	0.95-1.80	1.25	0.91-1.72
Number of hospitalizations in previous 60 days				
None	Ref			
1	1.25	0.97-1.61	1.27	0.99-1.64
>1	1.40	1.04-1.89	1.46	1.08-1.96
Age (per 1 year)	1.01	1.00-1.02	1.01	1.00-1.02
*At the onset or during treatment of iCDI*				
Gastric acid suppression	1.36	1.00-1.85	1.40	1.03-1.90
Cumulative fluoroquinolone exposure^a^	1.24	1.09-1.41	1.42	1.25-1.61
*Following completion of iCDI treatment*				
High risk antimicrobial^b^	2.95^c^	2.25-3.86	1.86	1.42-2.42
Fluoroquinolone	1.56^c^	1.63-2.08	0.86	0.64-1.15
IV vancomycin	1.45^c^	1.09-1.92	1.05	0.80-1.39

iCDI = initial episode of *C. difficile* infection.

^a^Cumulative fluoroquinolone exposure was modeled as a three node spline.

^b^High risk antimicrobials included all cephalosporins, clindamycin, and penicillins.

^c^Exposure at any time *t.*

## Discussion

We have identified eight discrete independent risk factors for recurrent CDI. Although some characteristics, such as age, cannot be altered, several of them constitute modifiable exposures. New gastric acid suppression and concomitant antimicrobial exposures were associated with increased hazards of developing recurrent CDI. Reducing these exposures could potentially decrease the risk of recurrent CDI. This may serve as yet another reason for institutions to engage in aggressive antimicrobial stewardship programs.

Prior investigations have reported advanced age, chronic renal insufficiency, elevated white blood cell count, low serum albumin, use of PPI and H2RB, as well as continued use of systemic antimicrobials to be important risk factors for rCDI [7,10-13,22]. Our results are in general agreement with these prior data. Gastric acid suppressors have garnered a particular interest with respect to their impact on iCDI and rCDI incidence. Specific to recurrent disease, a recent meta-analysis substantiated this concern, finding a more-than doubling of the risk of rCDI in the setting of these drugs [7]. At the same time, it is unclear whether both PPIs and H2RBs are associated with the risk of rCDI, or whether one is a more likely culprit than the other. For example a meta-analysis by Kwok and colleagues implicated PPIs but not H2RBs in a 2-fold rise of rCDI incidence [23]. Similarly, Tleyjeh et al. in a meta-analysis of 33 studies focusing specifically on H2RB exposure reported a smaller, albeit still significant, association between receiving H2RBs and development of CDI [24]. Both meta-analyses suggested that gastric protection in conjunction with antibiotic administration carries a higher risk of CDI development than exposure to PPIs or H2RBs alone [23,24]. In our study, we examined gastric acid suppressors as a single category because our prior work, including preliminary analyses for this study (data not shown), has consistently found no difference in the associations between these two classes of medications and CDI [17,18]. Whether gastric acid suppression is truly an independent risk factor for CDI or a marker for patients at risk for CDI remains unknown [6].

A large body of evidence also ties concomitant use of non-CDI antimicrobials to an increased risk of a recurrence [7,17,18,25]. We found that high-risk antimicrobials raise the risk for rCDI, particularly when administered after the completion of iCDI treatment. We have also confirmed previous findings that link exposure to such specific antimicrobials as IV vancomycin and fluoroquinolones to the risk for CDI incidence [17,18,26]. The BI/NAP1/027 strain has been associated with fluoroquinolone exposures, and may be more likely to cause rCDI than other strains of *C. difficile*[27]*.* Consequently, it is possible that fluoroquinolone exposure is a marker for CDI specifically due to this strain. For IV vancomycin, however, this association may represent not a causal relationship, but rather a marker for higher illness severity and, thus, confounding by indication.

We were also able to demonstrate the importance of timing of antimicrobial exposure after the end of CDI treatment. When modeled as time dependent variables, high-risk antimicrobials, fluoroquinolones, and IV vancomycin were all associated with rCDI. When modeled as time independent variables, the hazards of rCDI associated with high-risk antimicrobials dropped from 2.95 (2.25-3.86) to 1.86 (1.42-2.42), and fluoroquinolones and IV vancomycin were no longer associated with rCDI. Intuitively, this makes sense. An antimicrobial should not increase the risk of rCDI after CDI treatment has ended until the patient is exposed to the antimicrobial. Not modeling antimicrobials as time dependent variables after CDI treatment has ended dilutes the association with rCDI, since the days not on these drugs are included in the model.

A direct relationship between CO-HCFA status and iCDI and rCDI development is a newer finding [15]. Namely, the CO-HCFA designation of the iCDI episode is associated with at least a 25% and as much as a 2-fold increase in the risk of rCDI. A likely mechanism relates to the fact that CO-HCFA defines a population of patients who is likely sicker as evident by recent hospitalizations, and more likely to be exposed to antimicrobials. However, CO-HCFA CDI remained an independent risk factor when controlling for recent hospitalizations.

It is worth noting that the recurrence rate we observed in the current study is at the lower end of what has been reported previously. For example, a recent meta-analysis by Garey et al. examined the literature on risk factors for rCDI [7]. In the 12 studies meeting the inclusion criteria, the rates of recurrence ranged from 13% to 50%. More current data from randomized controlled trials suggest that CDI is likely to recur in approximately 25% of the patients treated for iCDI with vancomycin [8,9]. A potential explanation for the lower rCDI rate in our study compared to others is how cases of CDI were identified. Most stools submitted for *C. difficile* testing at the BJH microbiology laboratory come from inpatients and the emergency room. A minority of specimens come from outpatients or affiliated skilled nursing facilities. It is likely that milder cases of rCDI were missed because the patient did not require care in an emergency room or need to be admitted. Therefore, the rCDI in this study may consist of more clinically important episodes, occurring in sicker patients, many of whom required an admission or evaluation in the emergency department.

It is possible that patients who resided outside the St. Louis metropolitan area would not be likely to return to BJH for testing for a recurrence. To examine the impact of this potential loss to follow up, we performed a sensitivity analysis of rCDI risk factors by excluding all patients who resided beyond the greater St. Louis postal code. After excluding the 1230 (31.1%) patients with iCDI who met this criterion, the rCDI risk factors and their hazard ratios did not change appreciably (data not shown). This suggested that our results were not biased by including these patients.

Conversely, rCDI in randomized trials may be subject to a detection bias. Patients in trials are prospectively monitored for recurrent diarrhea and instructed to seek testing if it occurs. Even if the patient’s symptoms are not from CDI, the person may test positive for CDI as many patients continue to shed *C. difficile* in stool after cessation of CDI treatment [28].

Our study has some limitations. As a retrospective observational study it is prone to several forms of bias, most notably a selection bias. To mitigate this, we enrolled all consecutive patients meeting our enrollment criteria. To avoid misclassification of the main outcome variable, we applied a stringent case definition to CDI, which included a positive toxin assay. Although confounding is an issue with observational data, we adjusted for all the available relevant potential confounders in the regression model. However, the possibility of residual confounding remains. The biggest limitation, however, is its generalizability, since the data reflected patients and treatment patterns at an urban academic medical center with a large referral base, and may not have mirrored those of institutions with different characteristics or patients with iCDI diagnosed and managed completely in the outpatient setting. Additionally, many of the patients who resided outside the St. Louis metropolitan area may not have had their specimens retested at the BJH laboratory. After excluding these patients from the analysis as part of a sensitivity analysis, neither the rCDI hazard ratios nor the rCDI risk factors were majorly impacted in the overall cohort.

## Conclusion

In summary, we have demonstrated that a number of modifiable factors exist whose presence raises the risk for developing rCDI. Avoiding such exposures as non-CDI antimicrobial treatment and gastric acid suppressors may go a long way toward attenuating the burden of rCDI. On the other hand recognizing CO-HCFA and advanced age as predispositions to rCDI should serve patients and clinicians well by highlighting the importance of targeting these populations for more aggressive prevention efforts.

## Competing interests

MDZ reports that she has received research support from Optimer, and research and consulting support from ViroPharma and from Cubist. ERD reports that he has performed research for Viropharma and Merck and has served as a consultant for Merck, Becton-Dickinson, Optimer, Meridian, and Steris. All other authors report no conflicts of interest relevant to this article.

## Authors’ contributions

MDZ conceived of the study, participated in its design, interpretation of the analyses and drafting of the manuscript. KR participated in carrying out and coordinating the analyses and drafting of the manuscript. MO participated in carrying out the analyses and their interpretation, as well as drafting of the manuscript. YY was responsible for the integrity of the statistical analyses and for carrying them out, as well as drafting the manuscript. ERD participated in the design and oversight of the study, as well as drafting the manuscript. All authors read and approved the final manuscript.

## Pre-publication history

The pre-publication history for this paper can be accessed here:

http://www.biomedcentral.com/1471-2334/14/306/prepub

## References

[B1] Pépin J Valiquette L Alary ME Villemure P Pelletier A Forget K Pépin K Chouinard D *Clostridium difficile*-associated diarrhea in a region of Quebec from 1991 to 2003: a changing pattern of disease severity CMAJ 2004 14 466 472 10.1503/cmaj.1041104 15337727 PMC514643

[B2] McDonald LC Owings M Jernigan DB *Clostridium difficile* infection in patients discharged from US short-stay hospitals, 1996–2003 Emerg Infect Dis 2006 14 409 415 10.3201/eid1205.051064 16704777 PMC3291455

[B3] Zilberberg MD Shorr AF Kollef MH Increase in adult *Clostridium difficile*-related hospitalizations and case-fatality rate, United States, 2000–2005 Emerg Infect Dis 2008 14 929 931 10.3201/eid1406.071447 18507904 PMC2600276

[B4] Lucado, J. (Social & Scientific Systems), Gould, C. (CDC), and Elixhauser, A. (AHRQ) *Clostridium difficile* Infections (CDI) in Hospital Stays, 2009. HCUP Statistical Brief #124 2012 Agency for Healthcare Research and Quality, Rockville, MD. http://www.hcup-us.ahrq.gov/reports/statbriefs/sb124.jsp

[B5] Clements AC Magalhaes RJ Tatem AJ Paterson DL Riley TV *Clostridium difficile* PCR ribotype 027: assessing the risks of further worldwide spread Lancet Incect Dis 2010 14 395 404 10.1016/S1473-3099(10)70080-3 PMC7185771 20510280

[B6] Cohen SH Gerding DN Johnson S Kelly CP Loo VG McDonald LC Pepin J Wilcox MH Clinical practice guidelines for *Clostridium difficile* infection in adults: 2010 update by the society for healthcare epidemiology of America (SHEA) and the infectious diseases society of America (IDSA) Infect Control Hosp Epidemiol 2010 14 5 431 455 10.1086/651706 20307191

[B7] Garey KW Sethi S Yadav Y DuPont HL Meta-analysis to assess risk factors for recurrent *Clostridium difficile* infection J Hosp Infect 2008 14 298 304 10.1016/j.jhin.2008.08.012 18951661

[B8] Louie TJ Miller MA Mullane KM Weiss K Lentnek A Golan Y Gorbach S Sears P Shue YK OPT-80-003 Clinical Study Group Fidaxomicin versus vancomycin for *Clostridium difficile* infection N Engl J Med 2011 14 422 431 10.1056/NEJMoa0910812 21288078

[B9] Cornely OA Crook DW Esposito R Poirier A Somero MS Weiss K Sears P Gorbach S OPT-80-004 Clinical Study Group Fidaxomicin versus vancomycin for infection with *Clostridium difficile* in Europe, Canada, and the USA: a double-blind, non-inferiority, randomised controlled trial Lancet Infect Dis 2012 14 281 289 10.1016/S1473-3099(11)70374-7 22321770

[B10] Fekety R McFarland LV Surawicz CM Greenberg RN Elmer GW Mulligan ME Recurrent *Clostridium difficile* diarrhea: characteristics of and risk factors for patients enrolled in a prospective, randomized, double-blinded trial Clin Infect Dis 1997 14 324 333 10.1093/clinids/24.3.324 9114180

[B11] McFarland LV Surawicz CM Rubin M Fekety R Elmer GW Greenberg RN Recurrent *Clostridium difficile*: epidemiology and clinical characteristics Infect Control Hosp Epidemiol 1999 14 43 50 10.1086/501553 9927265

[B12] Cadle RM Mansouri MD Logan N Kudva DR Musher DM Association of proton-pump inhibitors with outcomes in *Clostridium difficile* colitis Am J Health Syst Pharm 2007 14 2359 2363 10.2146/ajhp060629 17989446

[B13] Kim JW Lee KL Jeong JB Kim BG Shin S Kim JS Jung H Son IS Proton pump inhibitors as a risk factor for recurrence of *Clostridium-difficile*-associated diarrhea World J Gastroenterol 2010 14 3573 3577 10.3748/wjg.v16.i28.3573 20653067 PMC2909558

[B14] Zilberberg MD Tabak YP Sievert DM Derby KG Johannes RS Sun X McDonald LC Using electronic health information to risk-stratify rates of *Clostridium difficile* infection in US hospitals Infect Control Hosp Epidemiol 2011 14 649 655 10.1086/660360 21666394

[B15] Eyre DW Walker AS Wylie D Dingle KE Griffiths D Finney J O'Connor L Vaughan A Crook DW Wilcox MH Peto TE Predictors of first recurrence of Clostridium difficile infection: implications for initial management Clin Infect Dis 2012 14 S77 S87 10.1093/cid/cis356 22752869 PMC3388024

[B16] McDonald LC Coignard B Dubberke E Song X Horan T Kutty PK Recommendations for surveillance of *Clostridium difficile*-associated disease Infect Control Hosp Epidemiol 2007 14 140 145 10.1086/511798 17265394

[B17] Dubberke ER Reske KA Yan Y Olsen MA McDonald LC Fraser VJ *Clostridium difficile*–associated disease in a setting of endemicity: identification of novel risk factors Clin Infect Dis 2007 14 1543 1549 10.1086/523582 18190314

[B18] Dubberke ER Yan Y Reske KA Butler AM Doherty J Pham V Fraser VJ Development and validation of a *Clostridium difficile* infection risk prediction model Infect Control Hosp Epidemiol 2011 14 360 366 10.1086/658944 21460487 PMC3649761

[B19] Hosmer DW Lemeshow S New York, NY Applied Logistic Regression 2000 2 New York, NY: John Wiley and Sons, Inc

[B20] Harrell FE Jr Margolis PA Gove S Mason KE Mulholland EK Lehmann D Muhe L Gatchallan S Eichenwald HF Development of a clinical prediction model for an ordinal outcome: the World Health Organization Multicentre Study of Clinical Signs and Etiological agents of Pneumonia, Sepsis and Meningitis in Young Infants. WHO/ARI Young Infant Multicentre Study Group Stat Med 1998 14 909 944 10.1002/(SICI)1097-0258(19980430)17:8<909::AID-SIM753>3.0.CO;2-O 9595619

[B21] SAS Copyright (c) 2002–2010 Cary, NC, USA: SAS Institute Inc

[B22] Kim YG Graham DY Jang BI Proton pump inhibitor use and recurrent *Clostridium difficile*-associated disease: a case–control analysis matched by propensity score J Clin Gastroenterol 2012 14 397 400 10.1097/MCG.0b013e3182431d78 22298089

[B23] Kwok CS Arthur AK Anibueze CI Singh S Cavallazzi R Loke YK Risk of *Clostridium difficile* infection with acid suppressing drugs and antibiotics: meta-analysis Am J Gastroenetrol 2012 14 1011 1019 10.1038/ajg.2012.108 22525304

[B24] Tleyjeh IM Abdulhak AB Riaz M Garbati MA Al-Tannir M Alasmari FA Alghamdi M Khan AR Erwin PJ Sutton AJ Baddour LM The association between histamine 2 receptor antagonist use and Clostridium difficile infection: a systematic review and meta-analysis PLoS One 2013 14 e56498 10.1371/journal.pone.0056498 23469173 PMC3587620

[B25] Mullane KM Miller MA Weiss K Lentnek A Golan Y Sears PS Shue YK Louie TJ Gorbach SL Efficacy of fidaxomicin versus vancomycin as therapy for *Clostridium difficile* infection in individuals taking concomitant antibiotics for other concurrent infections Clin Infect Dis 2011 14 440 447 10.1093/cid/cir404 21844027 PMC3156139

[B26] Pépin J Saheb N Coulombe MA Alary ME Corriveau MP Authier S Leblanc M Rivard G Bettez M Primeau V Nguyen M Jacob CE Lanthier L Emergence of fluoroquinolones as the predominant risk factor for *Clostridium difficile*-associated diarrhea: a cohort study during an epidemic in Quebec Clin Infect Dis 2005 14 1254 1260 10.1086/496986 16206099

[B27] Petrella LA Sambol SP Chenknis A Nagaro K Kean Y Sears PS Babakhani F Johnson S Gerding DN Decreased cure and increased recurrence rates for *Clostridium difficile* Infection caused by the epidemic *C. difficile* BI strain Clin Infect Dis 2012 [Epub ahead of print] 10.1093/cid/cis430 PMC3491778 22523271

[B28] Sethi AK Al-Nassir WN Nerandzic MM Bobulsky GS Donskey CJ Persistence of skin contamination and environmental shedding of *Clostridium difficile* during and after treatment of *C. difficile* infection Infect Control Hosp Epidemiol 2010 14 21 27 10.1086/649016 19929371

